# Independent factors associated with intracytoplasmic sperm injection outcomes in patients with complete azoospermia factor c microdeletions

**DOI:** 10.1093/hropen/hoae071

**Published:** 2024-11-26

**Authors:** Yangyi Fang, Zhe Zhang, Yinchu Cheng, Zhigao Huang, Jiayuan Pan, Zixuan Xue, Yidong Chen, Vera Y Chung, Li Zhang, Kai Hong

**Affiliations:** Department of Urology, Peking University Third Hospital, Beijing, China; Department of Urology, Peking University Third Hospital, Beijing, China; Department of Pharmacy, Peking University Third Hospital, Beijing, China; Department of Urology, Peking University Third Hospital, Beijing, China; Department of Urology, Peking University Third Hospital, Beijing, China; Department of Urology, Peking University Third Hospital, Beijing, China; Center for Reproductive Medicine, Department of Obstetrics and Gynecology, Peking University Third Hospital, Beijing, China; National Clinical Research Center for Obstetrics and Gynecology, Beijing, China; Key Laboratory of Assisted Reproduction (Peking University), Ministry of Education, Beijing, China; Department of Urology, Gleneagles Hong Kong Hospital, Hong Kong, China; Center for Reproductive Medicine, Department of Obstetrics and Gynecology, Peking University Third Hospital, Beijing, China; National Clinical Research Center for Obstetrics and Gynecology, Beijing, China; Key Laboratory of Assisted Reproduction (Peking University), Ministry of Education, Beijing, China; Department of Urology, Peking University Third Hospital, Beijing, China

**Keywords:** complete AZFc microdeletions, testicular sperm, ejaculated sperm, intracytoplasmic sperm injection, ICSI, cumulative live birth rate

## Abstract

**STUDY QUESTION:**

Which independent factors influence ICSI outcomes in patients with complete azoospermia factor c (AZFc) microdeletions?

**SUMMARY ANSWER:**

In patients with complete AZFc microdeletions, the sperm source, male LH, the type of infertility in women, and maternal age are the independent factors associated with ICSI outcomes.

**WHAT IS KNOWN ALREADY:**

AZF microdeletions are the second most prevalent factor contributing to infertility in men, with AZFc microdeletions being the most frequently affected locus, accounting for 60–70% of all cases. The primary clinical phenotypes are oligoasthenozoospermia and azoospermia in patients with complete AZFc microdeletions. These patients can achieve paternity through ICSI using either testicular (T-S) or ejaculated (E-S) spermatozoa. With aging in men with AZFc microdeletions, oligoasthenozoospermia or severe oligozoospermia may gradually progress to azoospermia.

**STUDY DESIGN, SIZE, DURATION:**

In this retrospective cohort study, the independent factors associated with the outcomes of 634 ICSI cycles in 634 couples with the transfer of 1005 embryos between February 2015 and December 2023 were evaluated. The analysis included 398 ICSI cycles in 398 couples using E-S and 236 ICSI cycles in 236 couples using T-S; all men had complete AZFc microdeletions.

**PARTICIPANTS/MATERIALS, SETTING, METHODS:**

The inclusion criteria were as follows: (i) men had complete AZFc microdeletions and (ii) the couple underwent ICSI treatment using T-S or E-S. The exclusion criteria were as follows: (i) cycles involving frozen–thawed oocytes; (ii) cycles in which all fresh embryos were frozen and not transferred; (iii) cycles lost to follow-up; and (iv) multiple ICSI cycles, apart from the first cycle for each couple. The primary outcome was the cumulative live birth rate per ICSI cycle, whereas the secondary outcomes were the clinical pregnancy rate per ICSI cycle, fertilization rate, and the no-embryo-suitable-for-transfer cycle rate (NESTR). Moreover, the maternal and neonatal outcomes were analyzed. Continuous variables showing non-normal distributions were expressed as median and interquartile range and were analyzed using the Kruskal–Wallis test. Categorical variables were expressed as percentages and were analyzed using the χ^2^ or Fisher’s exact test. Linear and logistic regression models were constructed to assess the independent factors associated with ICSI outcomes.

**MAIN RESULTS AND THE ROLE OF CHANCE:**

The T-S group exhibited inferior ICSI outcomes than the E-S group, marked by significantly reduced rates of cumulative live birth, clinical pregnancy, fertilization, high-quality embryos, blastocyst formation, and implantation, with higher NESTRs. However, the miscarriage rate and neonatal outcomes did not significantly differ between the groups. Multivariate linear regression analysis demonstrated that reduced fertilization rates were significantly associated with T-S use (adjusted β, −0.281; 95% CI, −0.332 to −0.229). Multivariate logistic regression demonstrated that increased NESTRs were significantly associated with T-S use (adjusted odds ratio (OR), 4.204; 95% CI, 2.340–7.691), along with uterine anomaly in women (adjusted OR, 2.853; 95% CI, 1.053–7.718), infertility in women with multiple etiologies (adjusted OR, 11.118; 95% CI, 2.034–66.508), and advanced maternal age (adjusted OR, 1.138; 95% CI, 1.029–1.263). The use of T-S (adjusted OR, 0.318; 95% CI, 0.188–0.528), uterine anomaly in women (adjusted OR, 0.263; 95% CI, 0.058–0.852), and increased maternal age (adjusted OR, 0.877; 95% CI, 0.801–0.958) were also associated with decreased clinical pregnancy rates per ICSI cycle. Likewise, lower cumulative live birth rates were associated with T-S use (adjusted OR, 0.273; 95% CI, 0.156–0.468), male LH levels (adjusted OR, 0.912; 95% CI, 0.837–0.990), uterine anomaly (adjusted OR, 0.101; 95% CI, 0.005–0.529), and increased maternal age (adjusted OR, 0.873; 95% CI, 0.795–0.958). No significant differences were observed in the maternal and neonatal outcomes between both groups.

**LIMITATIONS, REASONS FOR CAUTION:**

The study was based on a single-center, retrospective cohort design. The molecular diagnosis of AZFc microdeletions was reliant on loci sY254 and sY255 according to the European Academy of Andrology and European Molecular Genetics Quality Network guidelines. While our findings were based on the clinical phenotypes and laboratory parameters, the abnormalities in the genetic profiles of spermatogenesis and early embryonic development in patients between the T-S and E-S groups have not yet been elucidated.

**WIDER IMPLICATIONS OF THE FINDINGS:**

Our results offer important insights into the independent factors that influence ICSI outcomes in patients with complete AZFc microdeletions. ICSI using E-S is a more favorable therapeutic option for younger patients with AZFc microdeletions and with sperm present in their ejaculate. This study highlights a new direction to investigate the molecular and phenotypic differences between the T-S and E-S groups, which may contribute to the diagnosis and treatment of complete AZFc microdeletions.

**STUDY FUNDING/COMPETING INTEREST(S):**

This study was supported by Capital’s Funds for Health Improvement and Research (2022-2-4094), Beijing Natural Science Foundation (7232203, 7242164), National Key Research and Development Program (2021YFC2700200, 2023YFC2705600), National Natural Science Foundation of China (82301889), Peking University Third Hospital Innovation Transformation Fund (BYSYZHKC2023103), Peking University Third Hospital Clinical Cohort Construction Project (BYSYDL2023016), and Young Elite Scientists Sponsorship Program by CAST (2023QNRC001). None of the authors have any competing interests to declare.

**TRIAL REGISTRATION NUMBER:**

N/A.

WHAT DOES THIS MEAN FOR PATIENTS? Genetic AZFc microdeletions are small missing parts of the DNA in the Y chromosome, which can disrupt sperm production. This study elucidates the impact of different factors on the success of ICSI, a fertility treatment where a sperm is injected into an oocyte, in men with these AZFc microdeletions. The different factors include the type of sperm used (either testicular sperm extracted directly from the testes or ejaculated sperm), testes size, hormone levels, and maternal age. The origin of sperm, i.e. ejaculated or testicular, is one of the most important factors. However, it remains unclear whether it actually affects the success of ICSI. We found that compared with using ejaculated sperm, the use of testicular sperm resulted in lower success rates, i.e. lower fertilization rates and lower chances of a pregnancy and live birth. Thus, men having sperm in their semen are likely to experience better outcomes using ejaculated sperm for ICSI, rather than undergoing surgery to extract testicular sperm. Additionally, more studies are warranted to understand the biological differences between testicular and ejaculated sperm, which could help improve treatment options for men with AZFc microdeletions.

## Introduction

Infertility is a major public concern, affecting ∼15% of couples of reproductive age worldwide. Male factors contribute to approximately half of all infertility cases. Approximately 10–15% of men with infertility present with azoospermia (Giannouli *et al.*, 2004), and ∼60% of azoospermic cases result from testicular dysfunction (Ahmadi Rastegar *et al.*, 2015). Notably, at least 30% of infertility cases in men are attributed to genetic abnormalities (Goncalves *et al.*, 2017), with the prevalence ranging from 2% to 10% among men with infertility (Liu *et al.*, 2017).

Azoospermia factor (AZF) microdeletions, a genetic abnormality, occur in ∼1 in 4000 healthy men in specific regions on the long arm of the Y chromosome, termed AZF loci (AZFa, AZFb, and AZFc). They can lead to spermatogenic failure and affect 7.5% of men with infertility (Vogt *et al.*, 1996; Colaco and Modi, 2018). Among the AZF loci, AZFc is the most commonly deleted locus, occurring in ∼80% of cases. In contrast, AZFa, AZFb, and AZFb+c combined deletions occur in 0.5–4%, 1–5%, and 1–3% of all cases, respectively (Lange *et al.*, 2009). AZFa deletions lead to the complete absence of germ cells, causing severe spermatogenic failure. AZFb deletions disrupt meiosis, leading to spermatogenesis arrest at the spermatocyte stage (Krausz *et al.*, 2014). The palindromic structures and repetitive sequences of AZFc render it vulnerable to genomic rearrangements mediated by non-allelic homologous recombination during meiosis, leading to AZFc microdeletions (Kuroda-Kawaguchi *et al.*, 2001). Patients with AZFc microdeletions exhibit a range of clinical phenotypes, including oligoasthenozoospermia, severe oligozoospermia, and azoospermia (Rozen *et al.*, 2012).

For azoospermic individuals with AZFc microdeletions, testicular sperm (T-S) can be retrieved by microdissection testicular sperm extraction (microTESE), which demonstrates a high sperm retrieval rate ranging from 60% to 75% (Schlegel and Li, 1998; Klami *et al.*, 2018). For those wishing to father a child, ICSI is conducted using either T-S or ejaculated (E-S) spermatozoa. Nonetheless, men with AZFc microdeletions experience suboptimal ICSI outcomes, including lower rates of fertilization, (high-quality) embryo development, pregnancy, and live birth, along with higher rates of cycles with no-embryo-suitable-for-transfer (NESTR), when compared with men with a normal Y chromosome (Yamaguchi *et al.*, 2020; Zhang *et al.*, 2021a,b; Lan *et al.*, 2022). A meta-analysis further demonstrated that patients with AZFc microdeletions exhibit reduced rates of fertilization, clinical pregnancy, and live birth (Colaco and Modi, 2024). Notably, ICSI outcomes differ upon using T-S and E-S. Several studies have reported no significant differences in the clinical parameters of ICSIs between T-S and E-S groups in patients with AZFc microdeletions (Choi *et al.*, 2004; Zhu *et al.*, 2015; Goncalves *et al.*, 2017). However, Oates *et al.* reported that among patients with AZFc microdeletions, the T-S group demonstrated significantly poorer ICSI outcomes than the E-S group (Oates *et al.*, 2002).

To date, the independent factors that influence ICSI outcomes with different sperm sources (T-S vs E-S) in patients with complete AZFc microdeletions remain uncertain. This retrospective cohort study describes the independent factors of ICSI outcomes stratified by the E-S and T-S groups, with the aim of helping to optimize therapeutic strategies for these patients to improve ICSI outcomes.

## Materials and methods

### Participants

Ethical approval for this study was obtained from the local ethical review board (IRB00006761-M20223336). The records of patients with complete AZFc microdeletions treated with ICSI using T-S by microTESE and E-S at the Reproductive Medical Centre of Peking University Third Hospital between February 2015 and December 2023 were analyzed retrospectively. The World Health Organization Manual for the Laboratory Examination and Processing of Human Semen (fifth edition) defines azoospermia as no spermatozoa in the sediment of the centrifuged semen sample at least three times in each patient. Oligozoospermia is defined as a total sperm count <39 × 10^6^ or a sperm concentration <15 × 10^6^/ml per ejaculate according to the lower reference limits. Oligozoospermia is classified into mild (10–15 × 10^6^/ml of sperm), moderate (5–10 × 10^6^/ml), and severe groups (<5 × 10^6^/ml). Asthenozoospermia is defined as <32% progressive motility per ejaculate. Cryptozoospermia refers to no spermatozoa in fresh semen samples but the presence of sperm in centrifuged sediment (Mazzilli *et al.*, 2017; Yang *et al.*, 2018; Chen *et al.*, 2023). For all patients, peripheral blood was collected and subjected to sequence-tagged sites (STS) polymerase chain reaction to detect AZFc-specific STS markers (sY254 and sY255) and confirm complete AZFc microdeletions (Simoni *et al.*, 1999). Karyotyping analysis was conducted on peripheral blood samples to detect chromosomal abnormalities. Additionally, 1–2 months before microTESE, all male patients and female partners underwent a physical examination and assessment of sex hormone levels, including FSH, LH, and estradiol (E_2_) or testosterone (T).

The inclusion criteria were as follows: (1) men with complete AZFc microdeletions and (2) couples treated with ICSI using T-S or E-S. The exclusion criteria were as follows: (i) cycles involving frozen-thawed oocytes; (ii) cycles in which all embryos were frozen and not transferred; (iii) cycles lost to follow-up; and (iv) multiple ICSI cycles apart from the first cycle for each couple.

### Preparation of sperm retrieved from microTESE and semen for ICSI

All microTESE procedures were conducted under general or spinal anesthesia per established guidelines (Schlegel and Li, 1998), with minor modifications. In the testis, an incision was made along the scrotal midline. Subsequently, an incision was made in the tunica albuginea under an operating microscope (OPMI Vario/S88 System, Karl Zeiss, Oberkochen, Germany) to expose the testicular parenchyma while preserving the blood supply. The testicular parenchyma was examined at 12–24× magnification under the operating microscope. Thick, opaque seminiferous tubules were selected and minced with a pair of 1-ml sterile syringe needles into a homogeneous suspension in a dish containing G-MOPS-plus medium (Vitrolife, Gothenburg, Sweden). Sperm were identified using an inverted microscope (Nikon TE2000-U, Tokyo, Japan) at 200× magnification. Upon detection of sperm, the suspension was centrifuged at 450*g* for 10 min. The resultant pellet was transferred into droplets and overlaid with mineral oil in a dish, prepared for subsequent ICSI procedures. In cases where sperm retrieval did not align with the timing of ovarian stimulation of the woman, the sperm-containing cell suspension was combined with sperm cryopreservation solution (Vitrolife, Gothenburg, Sweden) in a 1:1 ratio and loaded into a 2-ml straw. The straw was maintained at room temperature for 10 min and immersed in a liquid nitrogen bath for 30 min. Subsequently, it was stored in a liquid nitrogen container. When needed, the frozen microTESE sperm were thawed in a 37 °C incubator for 15 min, mixed with 2 ml of washing medium, and centrifuged at 450*g* for 10 min. Subsequently, the frozen–thawed microTESE sperm was used for ICSI per the previously described protocol.

The swim-up technique was utilized for semen samples collected after 3–5 days of sexual abstinence via masturbation. Briefly, semen was combined with 2 ml of washing medium (G-MOPS-plus, Vitrolife) and centrifuged at 450*g* for 10 min. After removing the supernatant, the pellet was resuspended in 2 ml of the washing medium and centrifuged at 350*g* for 7 min. After removing the supernatant, 0.5–1 ml of washing medium was gently added to the pellet in a 4-ml tube. The tube was placed at a 45° angle and incubated at 37 °C for 15–30 min. After the swim-up procedure, 0.2–0.5 ml of the resultant swim-up solution was carefully pipetted and transferred into a fresh 4-ml tube for subsequent use. In the case of sperm obtained from patients with severe oligozoospermia, the pellet underwent two rounds of centrifugation before ICSI.

### Ovarian stimulation, oocyte retrieval, and ICSI

A gonadotropin-releasing hormone agonist or antagonist was used for ovarian stimulation in the female partners (Liu *et al.*, 2012). Follicular development was assessed through transvaginal ultrasonography and serum E_2_ measurement. When E_2_ concentration >500 pg/ml and at least one follicle measured 18 mm, 10 000 units of urinary hCG were administered (Serono, Aubonne, Switzerland). The following day, luteal phase support was initiated using 60 mg of progesterone (P) (Xianju Pharmacy, Zhejiang, China) after oocyte retrieval. Oocyte retrieval was conducted under the guidance of transvaginal ultrasound 36–38 h after hCG administration. Cumulus cells were separated from the oocytes 2-h post-retrieval via pipetting and hyaluronidase treatment (Type VIII; Sigma Chemical Company, St Louis, MO, USA). After selecting the appropriate sperm, ICSI was conducted using an injection pipette controlled by a micromanipulator on the day of oocyte retrieval (Day 0), as per the established protocol (Palermo *et al.*, 1992).

### Embryo transfer

Oocytes subjected to injection were incubated in appropriate culture media at 37 °C under an environment consisting of N_2_/CO_2_/O_2_ (90:5:5, v/v). The appearance of two pronuclei (2PN) within the embryo, 17–19 h after ICSI, indicated successful fertilization. The zygote was cultured in 25 µl of pre-equilibrated cleavage media (G1, Global HTF) and Quinn’s Advantage Cleavage Medium (Vitrolife) in the mentioned incubator until uterine transfer. Embryos were evaluated per the Society for Assisted Reproductive Technology scoring system (Racowsky *et al.*, 2011), based on cell number, fragmentation, and cell symmetry assessments on either Days 3 or 5 after ICSI. The highest-quality embryos were selected for uterine transfer. The remaining embryos were cultured to the blastocyst stage for cryopreservation or frozen directly. In cases of ovarian hyperstimulation syndrome or when other factors prevented embryo transfer, the embryos were similarly cultured to the blastocyst stage for cryopreservation or directly frozen for future frozen–thawed cycles. Clinical pregnancy was determined by detecting a gestational sac via transvaginal ultrasound examination on Day 35 after transfer. Miscarriage was defined as the loss of clinical pregnancy before 24 weeks of gestation.

### Outcomes and measurements

The primary outcome measure was the cumulative live birth rate per ICSI cycle, defined as the number of deliveries with at least one live birth resulting from one initiated ICSI cycle for fresh and/or frozen-thawed embryo transfer divided by the number of ICSI cycles. The outcome was measured until the first delivery with a live birth or use of all embryos, whichever occured first. The delivery of a singleton, twin, or other multiples was recorded as one delivery (Zegers-Hochschild *et al.*, 2017).

The secondary outcome measures were as follows: (1) fertilization rate, defined as the percentage of 2PN zygotes of the total number of MII oocytes; (2) high-quality embryo rate (HQER), calculated as the percentage of high-quality embryos among the total number of cleavage zygotes; (3) blastocyst formation rate (BFR), defined as the percentage of blastocysts formed relative to the total number of cultured embryos; (4) implantation rate, defined as the number of gestational sacs divided by the number of transferred embryos, with at most two embryos transferred per woman; (5) NESTR, defined as no embryo suitable for transfer cycles divided by the total number of ICSI cycles; (6) clinical pregnancy rate per ICSI cycle, defined as the percentage of clinical pregnancies (detected under ultrasonography of one or more gestational sacs) relative to the total number of ICSI cycles; and (7) miscarriage rate, calculated as the number of miscarriage cycles divided by the number of clinical pregnancies.

Maternal outcomes were characterized by maternal complications, including gestational hypertension, gestational diabetes, premature birth (defined as a gestational age between 28 and 36 weeks at delivery), and cesarean delivery during gestation. Neonatal outcomes were assessed based on neonatal complications, including low birth weight (<2500 g), very low birth weight (<1500 g), and macrosomia (>4000 g) during the perinatal period (Wang *et al.*, 2021). The neonates were divided into two subgroups as follows: singleton and twin live birth deliveries, with separate statistical analyses for each group.

### Statistical analysis

Continuous variables with normal distributions are reported as mean±SD and were compared between the groups using Student’s *t*-test. Variables with non-normal distributions are reported as median (IQR) and were analyzed using the Kruskal–Wallis test. Categorical variables are presented as percentages, with between-group differences evaluated using the χ^2^ test or Fisher’s exact test. A multivariable linear regression analysis was performed to evaluate the independent factors that influenced the fertilization rate. Additionally, multivariable logistic regression models were utilized to identify the independent factors associated with the cumulative live birth rate per ICSI cycle, clinical pregnancy rate per ICSI cycle, and NESTR. Findings from both linear and logistic regression analyses were reported separately. The linear regression results are expressed as adjusted beta coefficients (a-β), whereas the logistic regression outcomes are expressed as adjusted odds ratios (a-OR), each accompanied by a 95% CI. All data were analyzed by SPSS software version 16.0 (IBM Corp., NY, USA) and R version 4.0.2 (The R Foundation for Statistical Computing, Vienna, Austria). Statistical significance was defined as a two-sided *P*-value <0.05.

## Results

### Clinical characteristics

**Figure 1.**
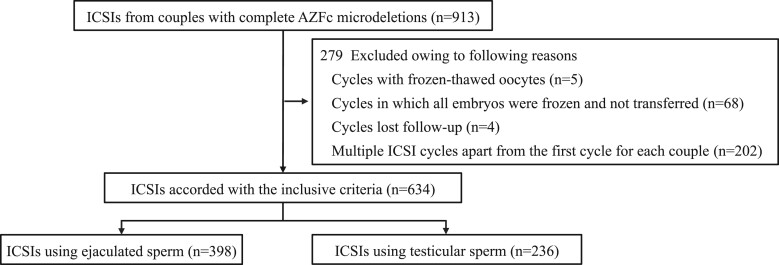
**Flow chart of the patients**. AZFc, azoospermic factor c.

A total of 634 ICSI cycles were enrolled, and the patients were categorized into the E-S (n = 398) and T-S (n = 236) groups (Fig. 1). Table 1 summarizes the clinical characteristics of all patients. The T-S group demonstrated higher male FSH and LH levels and lower total testicular volume (defined as the total volume of left and right testes), compared with the E-S group (FSH, 16.10 [11.20, 22.88] vs 9.27 [6.28, 12.70], *P* < 0.001; LH, 6.56 [4.70, 9.01] vs 4.46 [3.60, 7.71], *P* < 0.001; and total testicular volume, 17 [12.5, 24] vs 24 [20, 24], *P* < 0.001). The groups did not differ concerning the age, BMI, and T levels.

**Table 1.** Clinical characteristics of participants.

	Total cycles (n = 634)	ICSI cycles using ejaculated sperm (n = 398)	ICSI cycles using testicular sperm (n = 236)	*P-*value
Male clinical characteristics				
Age (years) median (IQR)^a^	31 (28, 33)	30 (28, 33)	31 (28, 34)	0.088
BMI (kg/m^2^) median (IQR)^a^	24.69 (22.60, 27.40)	24.49 (22.54, 27.36)	24.93 (22.60, 27.44)	0.366
Total testicular volume (ml), median (IQR)^a^	20 (16, 24)	24 (20, 24)	17 (12.5, 24)	**<0.001**
FSH (mIU/ml), median (IQR)^a^	11.2 (7.61, 16.90)	9.27 (6.28, 12.70)	16.10 (11.20, 22.88)	**<0.001**
LH (mIU/ml), median (IQR)^a^	5.34 (3.60, 7.71)	4.46 (3.23, 6.78)	6.56 (4.70, 9.01)	**<0.001**
T (nmol/l), median (IQR)^a^	10.10 (7.73, 12.98)	10.30 (7.88, 13.45)	9.60 (7.59, 12.60)	0.258
Female clinical characteristics				
Age (years) median (IQR)^a^	30 (27, 32)	29.5 (27, 32)	30 (27, 33)	0.742
BMI (kg/m^2^) median (IQR)^a^	21.50 (20.00, 24.20)	21.80 (20.00, 24.60)	21.40 (19.90, 23.55)	0.059
FSH (mIU/ml), median (IQR)^a^	5.80 (4.11, 7.26)	5.97 (4.72, 7.39)	5.32 (2.07, 7.14)	**0.008**
LH (mIU/ml), median (IQR)^a^	3.39 (1.90, 4.74)	3.72 (2.26, 4.79)	2.83 (1.42, 4.62)	**<0.001**
E_2_ (pmol/l), median (IQR)^a^	146 (109, 191)	155 (120, 202)	131 (96.8, 173.1)	**<0.001**
P (nmol/l), median (IQR)^a^	1.10 (0.76, 1.46)	1.07 (0.76, 1.45)	1.15 (0.76, 1.47)	0.276
PRL (ng/ml), median (IQR)^a^	12.60 (9.57, 17.70)	12.60 (9.98, 17.70)	12.65 (9.27, 18.10)	0.952
AFC, median (IQR)^a^	12 (9, 16)	13 (9.75, 16)	11 (9, 15)	**0.015**
ET-hCG (mm), median (IQR)^a^	11 (10, 12)	11 (10, 12)	11 (10, 12)	0.974
Type of infertility				
Normal uterus, % (n)^b^	82.96 (526/634)	81.41 (324/398)	85.60 (202/236)	0.175
Polycystic ovary syndrome, % (n)^b^	8.68 (55/634)	9.55 (38/398)	7.20 (17/236)	0.311
Endometriosis, % (n)^b^	1.58 (10/634)	1.76 (7/398)	1.27 (3/236)	0.751
Uterine anomalies, % (n)^b^	5.21 (33/634)	5.28 (21/398)	5.08 (12/236)	0.916
More than on etiology, % (n)	1.58 (10/634)	2.01 (8/398)	0.85 (2/236)	0.336
Other parameters				
Duration of infertility (years), median (IQR)^a^	3 (2, 5)	3 (2, 5)	3 (2, 4)	0.896
PGT cycles, % (n)^b^	20.50 (130/634)	22.11 (88/398)	17.80 (42/236)	0.193
ICSI cycles with fresh sperm, % (n)	92.43 (586/634)	100 (398/398)	79.66 (188/236)	
ICSI cycles with frozen-thawed sperm, % (n)	7.57 (48/634)	0	20.34 (48/236)	

Results are reported as median and IQR.

a The *P*-value was calculated using the Kruskal–Wallis test.

b The *P*-value was calculated using the chi-squared test (χ^2^ test).

P-values in bold are significant (P < 0.05).

IQR, interquartile range; T, testosterone; E_2_, estrogen; P, progesterone; PRL, pituitary prolactin; AFC, antral follicle count; ET-hCG, endometrial thickness on the day of human chorionic gonadotrophin administration; PGT, preimplantation genetic testing.

Fertility in women was classified into five types as follows: (1) normal uterus, including normal fertility and tubal factors (though possibly advanced age); (2) polycystic ovary syndrome (PCOS); (3) endometriosis; (4) uterine anomalies, including uterine fibroids, adenomyosis, endometritis, and endometrial polyps; or (5) more than one etiology (PCOS or endometriosis, combined with uterine anomalies). In the women, FSH, LH, and E_2_ levels as well as AFC were within normal limits in both groups, despite significant differences. The duration of infertility and proportion being PGT cycles, as well as the female age, BMI, P, and prolactin (PRL) levels, endometrial thickness on the day of hCG administration (ET-hCG), type of female infertility did not differ significantly between the two groups.

### Sperm analysis of the E-S group

**Figure 2.**
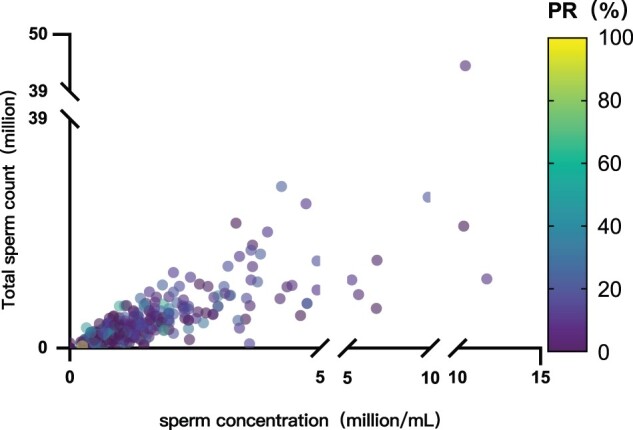
**Sperm analysis of the E-S group**. The bubble chart represents the distribution of sperm analysis parameters, and each dot represents an independent sperm sample. The *X*-axis denotes sperm concentration (million/ml), the *Y*-axis denotes the total sperm count (million), and the color scale on the right denotes the progressive motility rate (PR%). The color gradient ranges from purple (0%) to yellow (100%), illustrating the varying levels of sperm motility. E-S, ejaculated sperm.

Figure 2 illustrates the semen parameters of the E-S group. Of all patients, 74 were diagnosed with cryptozoospermia and 324 were diagnosed with oligozoospermia or asthenozoospermia in the E-S group. When classified by severity, there were 3, 4, and 317 patients in the mild, moderate, and severe oligozoospermia groups, respectively. The median sperm total count, concentration, and progressive motility (PR%) were 2.51 million (IQR, 0.95–4.91), 0.79 million/ml (IQR, 0.00–1.68), and 5.94% (IQR, 0.00–22.00), respectively.

### ICSI–ET outcomes

Table 2 summarizes the ICSI–ET outcomes. Compared with the E-S group, the T-S group demonstrated poorer clinical outcomes, with lower rates of fertilization (31.61% vs 59.81%, *P* < 0.001), HQER (45.75% vs 57.61%, *P* < 0.001), BFR (22.40% vs 34.49%, *P* < 0.001), implantation rates (23.97 vs 32.40, *P* = 0.008), clinical pregnancy rates per ICSI cycle (29.24% vs 49.75%, *P* < 0.001), and cumulative live birth rates per ICSI cycle (20.34% vs 44.47%, *P* < 0.001), compared with the E-S group. Additionally, NESTR was higher in the T-S group than in the E-S group (31.78% vs 14.57%, *P* < 0.001). The miscarriage rate did not differ significantly between the groups (14.14% vs 15.94%, *P* = 0.715).

**Table 2.** ICSI and neonatal outcomes in subgroups.

	ICSI cycles using ejaculated sperm (n = 398)	ICSI cycles using testicular sperm (n = 236)	*P*-value
ICSI outcome			
Fertilization rate, % (n)	59.81 (2695/4506)	31.61 (852/2695)	**<0.001**
HQER, % (n)	57.61 (1760/3055)	45.75 (555/1213)	**<0.001**
BFR, % (n)	34.49 (515/1493)	22.40 (123/549)	**<0.001**
NESTR, % (n)	14.57(58/398)	31.78 (75/236)	**<0.001**
Implantation rate, % (n)	32.40 (231/713)	23.97 (70/292)	**0.008**
Clinical pregnancy rate per ICSI cycle, % (n)	49.75 (198/398)	29.24 (69/236)	**<0.001**
Cumulative live birth rate per ICSI cycle, % (n)	44.47 (177/398)	20.34 (48/236)	**<0.001**
Miscarriage rate, % (n)	14.14 (28/198)	15.94(11/69)	0.715
Singleton live birth delivery cycle, % (n)	85.88 (152/177)	93.75(45/48)	0.143
Maternal outcomes for singleton births			
Gestational hypertension, % (n)	0.66 (1/152)	0	
Gestational diabetes, % (n)	3.95 (6/152)	6.67 (3/45)	0.443
Preterm birth ≤36 weeks, % (n)	7.24 (11/152)	6.67 (3/45)	0.896
Cesarean delivery, % (n)	59.87 (91/152)	57.78 (26/45)	0.802
Neonatal outcomes for singletons			
Very low birth weight (<1500 g), % (n)	0.66 (1/152)	0	–
Low birth weight (<2500 g), % (n)	3.29 (5/152)	8.89 (4/45)	0.214
Macrosomia (>4000 g), % (n)	6.58 (10/152)	4.44 (2/45)	0.737
Sex ratio at birth in non-PGT cycles, (male/female)	(59/54)	(19/19)	0.813
Twin live birth delivery cycle, % (n)	14.12(25/177)	6.25(3/48)	0.143
Maternal outcomes for twin births			
Gestational hypertension, % (n)	4.00 (1/25)	0	–
Gestational diabetes, % (n)	8.00 (2/25)	0	–
Preterm birth ≤36 weeks, % (n)	44.00 (11/25)	100.00 (3/3)	0.222
Cesarean delivery, % (n)	84.00(21/25)	100.00 (3/3)	1.000
Neonatal outcomes for twin pairs			
Very low birth weight (<1500 g), % (n)	0	0	–
Low birth weight (<2500 g), % (n)	24.00 (12/50)	83.33(5/6)	**0.008**
Macrosomia (>4000 g), % (n)	0	0	–
Sex ratio at birth in non-PGT cycles, (male/female)	(30/20)	(2/4)	0.385

Results are reported as percentages. The *P*-value was calculated using the chi-squared test (χ^2^ test). *P*-values in bold are significant (*P *<* *0.05).

HQER, high-quality embryo rate; BFR, blastocyst formation rate; NESTR, no-embryo-suitable-for-transfer cycle rate; PGT, preimplantation genetic testing.

### Maternal and neonatal outcomes

Maternal outcomes, including the proportions with gestational hypertension, gestational diabetes, premature birth, and cesarean delivery did not differ significantly between the groups. A total of 253 newborns were delivered, including 202 from the E-S group and 51 from the T-S group. The rates of singleton (85.88% vs 93.75%) or twin (14.12% vs 6.25%) live births did not differ significantly between the groups. However, concerning neonatal outcomes for twin births, the incidence of low birth weight was 24% (12/50) in the E-S group and 83.33% (5/6) in the T-S group.

### Independent factors associated with ICSI outcomes

**Figure 3.**
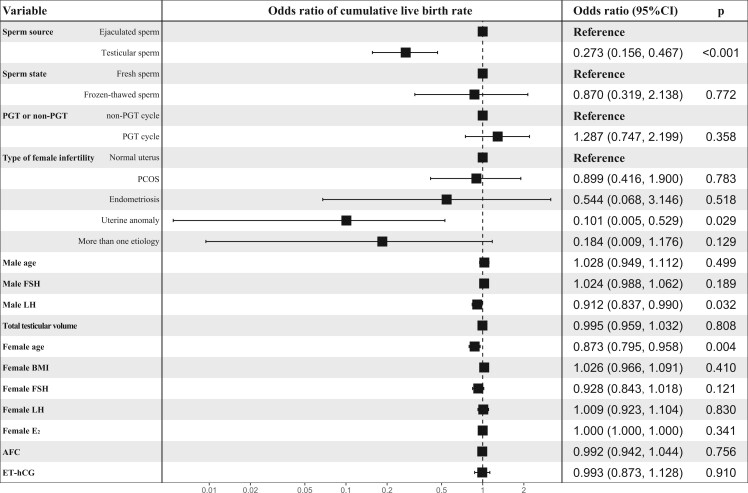
**Analysis of independent factors associated with the rates of cumulative live birth for patients with complete AZFc microdeletions**. Variables include the sperm source, sperm state, PGT or non-PGT cycle, age in men, male FSH levels, male LH levels, testicular volume, the type of infertility in women, maternal age, female BMI, female FSH levels, female LH levels, female E_2_ levels, AFC, and ET-hCG. AZFc, azoospermic factor c; PGT, preimplantation genetic testing; E2, estrogen; AFC, antral follicle count; ET-hCG, endometrial thickness on the day of human chorionic gonadotrophin administration.

**Figure 4.**
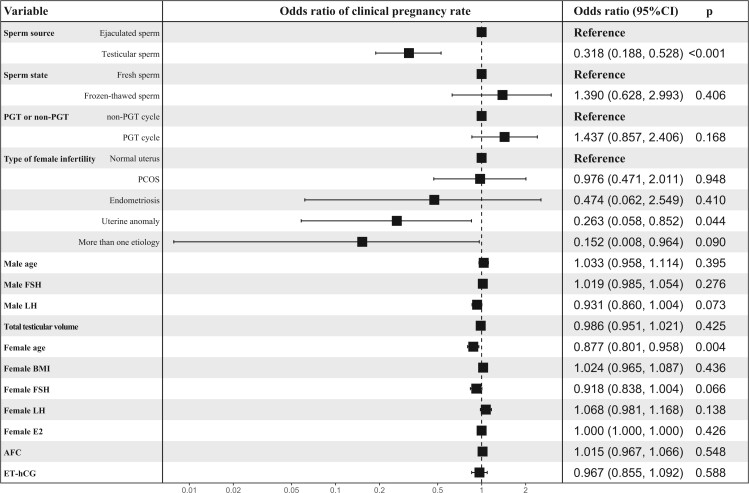
**Analysis of independent factors associated with the rates of clinical pregnancy per ICSI cycle for patients with complete AZFc microdeletions**. Variables include the sperm source, sperm state, PGT or non-PGT cycle, age in men, male FSH levels, male LH levels, testicular volume, the type of infertility in women, maternal age, female BMI, female FSH levels, female LH levels, female E_2_ levels, AFC, and ET-hCG. AZFc, azoospermic factor c; PGT, preimplantation genetic testing; E2, estrogen; AFC, antral follicle count; ET-hCG, endometrial thickness on the day of human chorionic gonadotrophin administration.

Multivariate logistic regression analysis suggested that factors, including the use of T-S (a-OR, 0.273; 95% CI, 0.156–0.468), infertility caused by uterine anomaly (a-OR, 0.101; 95% CI, 0.005–0.529), male LH levels (a-OR, 0.912; 95% CI, 0.837–0.990), and increased maternal age (a-OR, 0.873; 95% CI, 0.795–0.958) were independently associated with a low cumulative live birth rate (Fig. 3). Similarly, factors, including T-S use (a-OR, 0.318; 95% CI, 0.188–0.528), uterine anomaly in women (a-OR, 0.263; 95% CI, 0.058–0.852), and advanced maternal age (a-OR, 0.877; 95% CI, 0.801–0.958) were associated with decreased odds of clinical pregnancy rate per ICSI cycle (Fig. 4).

**Figure 5.**
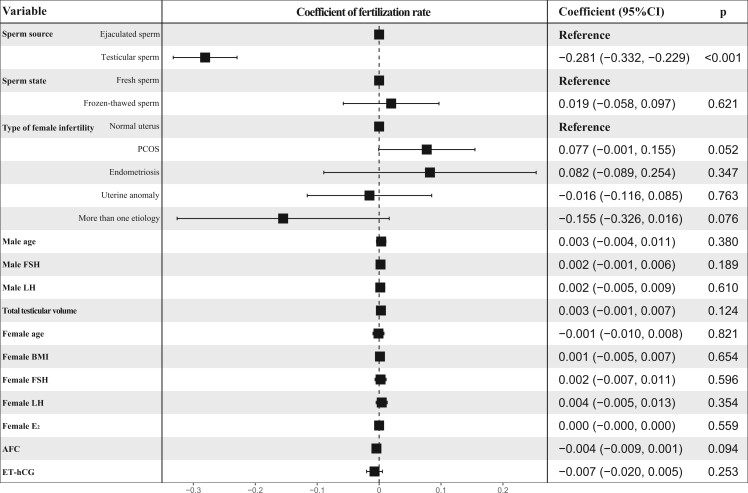
**Analysis of independent factors associated with the rates of fertilization for patients with complete AZFc microdeletions**. Variables include the sperm source, sperm state, age in men, male FSH levels, male LH levels, testicular volume, the type of infertility in women, maternal age, female BMI, female FSH levels, female LH levels, female E_2_ levels, AFC, and ET-hCG. AZFc, azoospermic factor c; E2, estrogen; AFC, antral follicle count; ET-hCG, endometrial thickness on the day of human chorionic gonadotrophin administration.

**Figure 6.**
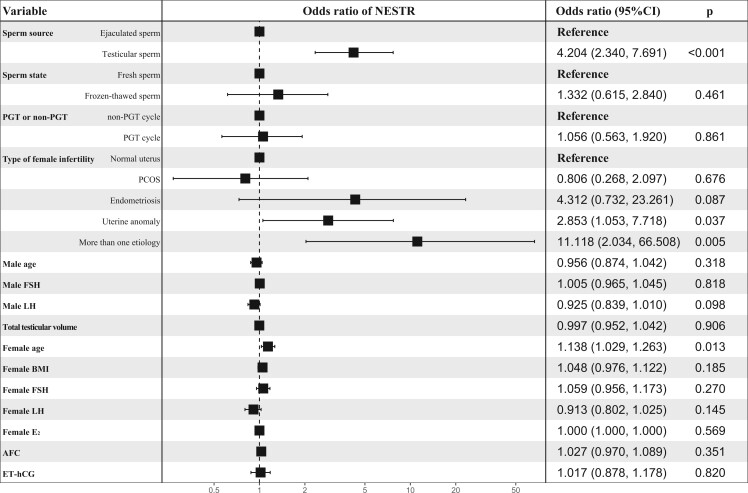
**Analysis of independent factors associated with the rates of NESTR for patients with complete AZFc microdeletions**. Variables include the sperm source, sperm state, PGT or non-PGT cycle, age in men, male FSH levels, male LH levels, testicular volume, the type of infertility in women, maternal age, female BMI, female FSH levels, female LH levels, female E_2_ levels, AFC, and ET-hCG. NESTR, no embryo suitable for transfer cycle rate; AZFc, azoospermic factor c; PGT, preimplantation genetic testing; E2, estrogen; AFC, antral follicle count; ET-hCG, endometrial thickness on the day of human chorionic gonadotrophin administration.

Multivariate linear regression analysis demonstrated that reduced fertilization rate was only associated with T-S use (adjusted β, −0.281; 95% CI, −0.332 to −0.229) (Fig. 5). However, T-S use (a-OR, 4.204; 95% CI, 2.340–7.691), uterine anomaly in women (a-OR, 2.853; 95% CI, 1.053–7.718), infertility in women caused by more than one etiology (a-OR, 11.118; 95% CI, 2.034–66.508), and advanced maternal age (a-OR, 1.138; 95% CI, 1.029–1.263) were the independent factors associated with increased odds of NESTR (Fig. 6).

## Discussion

This retrospective study includes the largest sample size to date to analyze the independent factors associated with ICSI outcomes in patients with complete AZFc microdeletions. Outcomes of 634 ICSI cycles from 634 couples, including 398 ICSI cycles with E-S from 398 couples and 236 ICSI cycles with T-S from 236 couples, demonstrated that T-S, low male LH levels, uterine anomalies, female infertility of multiple etiologies, and advanced maternal age were independently associated with adverse ICSI outcomes. To the best of our knowledge, this is the first study to highlight sperm source as an independent factor of ICSI outcomes. Meanwhile, the T-S group demonstrated poorer ICSI outcomes with lower rates of fertilization, HQER, BFR, implantation, clinical pregnancy per ICSI cycle, as well as lower cumulative live birth rates and higher rates of cycles with no embryo suitable for transfer, compared with the E-S group.

T-S was independently associated with adverse ICSI outcomes in patients with complete AZFc microdeletions. Oates *et al.* demonstrated poor outcomes of 48 ICSI cycles with lower rates of fertilization (36% vs 64%), pregnancy per ICSI cycle (14% vs 47%), and cumulative live birth (14% vs 47%) in the T-S group, compared with the E-S group (Oates *et al.*, 2002), consistent with our results. Nevertheless, pregnancy and delivery outcomes are comparable in the T-S and E-S groups (Choi *et al.*, 2004; Zhu *et al.*, 2015; Goncalves *et al.*, 2017). The discrepancies in ICSI outcome parameters between previous studies and the current study are likely attributed to differences in inclusion criteria (AZFc microdeletions vs complete AZFc microdeletions) and diverse sample sizes (27, 57, and 67 vs 836 cycles). The current study includes the largest sample size; thus, our results offer a more objective and reliable assessment of sperm source as an independent factor that influences ICSI outcomes in patients with complete AZFc microdeletions.

Interestingly, the T-S group demonstrated higher NESTR than the E-S group, which was a novel finding. The T-S group demonstrated smaller testicular volume and a higher level of FSH than the E-S group, likely indicating more severe spermatogenic dysfunction. This dysfunction may contribute to more severe impaired embryonic development, potentially causing a higher NESTR in the T-S group. Furthermore, our results suggested that an increased cumulative live birth rate was associated with a low male LH level. Hashimoto *et al.* demonstrated significant increases in LH levels upon administering clomiphene citrate in healthy men (Hashimoto *et al.*, 1975). Clomiphene citrate therapy can improve semen parameters through the central modulation of LH levels in men with infertility (Cannarella *et al.*, 2019; Jiang *et al.*, 2022). Therefore, we hypothesize that clomiphene citrate treatment may benefit patients with oligozoospermia along with complete AZFc microdeletions by improving ICSI outcomes. However, further studies are needed to substantiate its efficacy and elucidate the underlying mechanisms.

Men with oligozoospermia or fertile men with AZFc microdeletions may gradually develop azoospermia with aging (Simoni *et al.*, 1997; Chang *et al.*, 1999; Deng *et al.*, 2023). Men with complete AZFc microdeletions should consider ICSI or sperm cryopreservation at a younger age, while spermatozoa are still present in the semen, to optimize ICSI success. In contrast, with aging, patients with azoospermia along with complete AZFc microdeletions may have to undergo invasive microTESE procedures and have lower chances of biological fatherhood than patients with oligozoospermia (20.34% vs 44.47%).

This study has some limitations. First, the mechanisms underlying poorer ICSI outcomes in patients with complete AZFc microdeletions along with azoospermia were not compared with patients presenting with oligozoospermia. Researchers have elucidated the complete sequencing of the Y chromosome by the Telomere-to-Telomere consortium (Rhie *et al.*, 2023). This advancement enhances our understanding of the genetic differences between the groups, thus improving diagnosis and treatment. Second, the differences in early embryonic development at the molecular level were not compared between the two groups. A comprehensive study of the molecular mechanism underlying early embryos from the T-S and E-S groups could elucidate the adverse effects of Y-based genetic defects and spermatogenesis abnormalities on early embryonic development, particularly for the T-S group. A profound investigation into these molecular mechanisms may highlight novel strategies for improving early embryonic development outcomes.

This cohort study describes the independent factors that influence ICSI outcomes in Chinese patients with complete AZFc microdeletions. The results highlight the importance of early intervention, suggesting that these patients should either undergo ICSI using E-S or opt for sperm cryopreservation at a younger age to enhance their likelihood of achieving biological parenthood. In conclusion, our results not only identified novel independent factors of ICSI outcomes but also guide andrologists, particularly in determining the optimal timing for ICSI treatments and directing patients toward the most effective therapeutic options to optimize ICSI outcomes.

## Data Availability

The data supporting this article were provided by the Reproductive Medical Center of Peking University Third Hospital. Access to the data can be granted upon request to the corresponding authors, subject to approval from the Peking University Third Hospital.
